# Increasing Chlamydia and Gonorrhea Infections among Female Juveniles: The Need for Collaboration to Improve Treatment

**DOI:** 10.7759/cureus.8446

**Published:** 2020-06-04

**Authors:** Michael Neeki, Fanglong Dong, Lydia Lowe, Melinda Cerda, Carlos Peace, Kristina Roloff, Carol Lee

**Affiliations:** 1 Emergency Medicine, Arrowhead Regional Medical Center, Colton, USA; 2 Probation Department, San Bernardino County Department of Probation, San Bernardino, USA; 3 Research Emergency Medicine, Arrowhead Regional Medical Center, Colton, USA; 4 Epidemiology and Public Health, San Bernardino County Department of Probation, San Bernardino, USA; 5 Obstetrics and Gynecology, Arrowhead Regional Medical Center, Colton, USA

**Keywords:** sexually transmitted diseases, juvenile detention, screening, public health

## Abstract

Background: Youth in juvenile detention centers are at a high risk for sexually transmitted disease (STD). The current study assesses the trends of chlamydia and gonorrhea (GC) infections and treatment among females within a single county’s juvenile correctional facilities.

Methods: This is a retrospective review of aggregate data of female adolescents between 12 and 18 years old who underwent STD screening from 2009 to 2016 in San Bernardino County.

Results: Chlamydia infections among adolescent females increased from 11.8% in 2009 to 17.0% in 2016 (p = 0.0002), and GC infections increased from 1.3% in 2009 to 6.0% in 2016 (p < 0.0001). Treatment rates of chlamydia were stable, ranging from 66.7% to 70.8% of positive female adolescents were treated between 2009 and 2016 (p=0.1752). The treatment rate for GC increased from 33% in 2009 to 78.3% in 2016, but annual trends were not statistically significant (p=0.8419).

Conclusions: Chlamydia and GC infections among female adolescents discovered during routine screening upon booking into a county juvenile detention system increased over the study time period. Effective collaboration between public health and various community organizations is needed to improve awareness and prevention of STDs amongst at-risk adolescents.

## Introduction

Chlamydia trachomatis (CT) is the most commonly diagnosed sexually transmitted disease (STD) among adolescents incarcerated in juvenile correctional facilities in the United States [1]. The prevalence of CT infections within the past decade among incarcerated adolescents exceeds 14% for females and 6% for males [1,2]. Similarly, the rates of gonorrhea (GC) infections among incarcerated adolescents was reported at 4.2% for females and 1.6% for males under 20 years of age [3]. Detained youth may not have access to contraception or education on risky sexual behavior outside of the correctional facility, and may be more likely to engage in risky sexual behaviors, resulting in the increase of CT and GC infections. Furthermore, discrepancies may exist between self-reporting of sexual behavior and findings from laboratory testing [4]. These factors suggest the importance of universal screening amongst juvenile detainees in order to provide the most accurate representation of STD infections within the juvenile correctional system.

The Centers for Disease Control and Prevention (CDC) made nationwide recommendations for universal CT and GC screening in correctional facilities upon intake for females <35 years old and males <30 years old [5]. California initiated the state-funded Chlamydia Screening Project (CLaSP) in 2002 at selected institutions with an aim to increase CT screening and timeliness of treatment among incarcerated youth [6]. Timeliness of screening is important to ensure that all detained adolescents are screened before they are released from the correctional facilities [7]. Screening programs typically include prevention and disease education. Wiehe et al. found that youth with follow up of one year post-detection had not been re-infected, which suggests that STD screening may have a lasting benefit to the communities into which the detained youth are released [8]. 

Burghardt et al. recently reported a significant decreasing trend of CT infections upon booking under CLaSP within several California juvenile correctional facilities [9]. In addition, Barry et al. indicated that there were higher rates of STDs within the female detainee population than the male detainee population, and thus should be given priority in screening [10,11]. In light of conflicting reports, the current study aims to assess the rate of CT and GC infections at the time of booking among female adolescents in juvenile correctional facilities located in San Bernardino County, California.

## Materials and methods

We conducted a review of aggregate data of female adolescents between 12 and 18 years old that underwent STD screening for chlamydia and GC from 2009 to 2016 at the time of booking into juvenile correctional facilities located in San Bernardino County. San Bernardino County is the largest county geographically in the contiguous United States and has two juvenile correctional facilities managed by the San Bernardino County Department of Probation.

Data included the number of female adolescents screened upon booking, the rate of CT and GC infections, and the number of female adolescents who received treatment. The rate of infections was defined as the number of adolescents testing positive for CT or GC divided by the number of adolescents booked. Screening coverage was defined as the number of adolescents screened divided by the total number of adolescents booked. Treatment coverage was defined as the number treated divided by the total number of adolescents with infections in a given year. Female adolescents who tested positive and were still in custody were notified and treated. Those who were released prior to notification of a positive test result were included as untreated.

STD screening was conducted via universal CT and GC urine nucleic acid amplification-based testing. Patients reserved the right to refuse testing and minor consent for medical care was carried out in accordance with the California Family Code, Section 6926. All statistical analyses were conducted using the SAS software for Windows version 9.3 (SAS Institute Inc., Cary, North Carolina). Descriptive statistics were presented as frequencies and proportions. Cochran-Armitage tests were conducted to assess the trend of positive screening and treatment. All statistical analyses were two-sided and p-values less than 0.05 were considered to be statistically significant.

## Results

**Figure 1:**
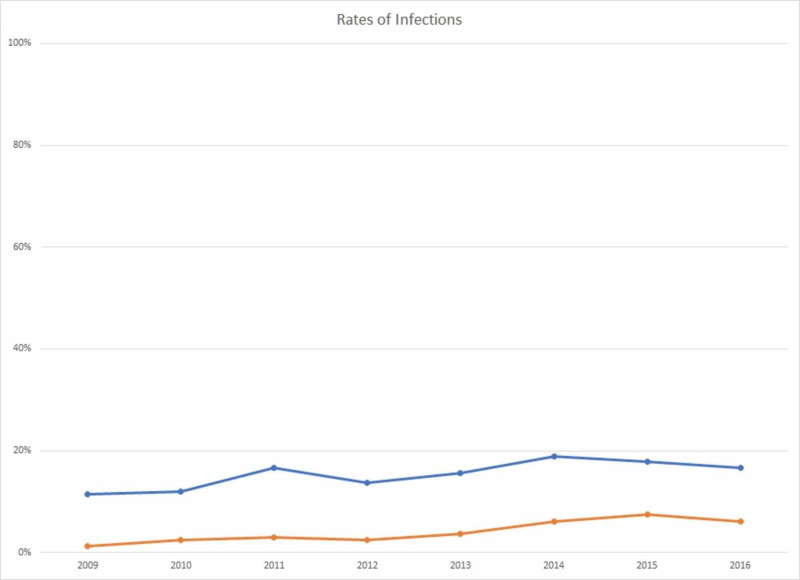
Rates of Chlamydia trachomatis and GC infections in female adolescent juveniles from 2009 to 2016 CT=Chlamydia; GC=Gonorrhea

**Figure 2:**
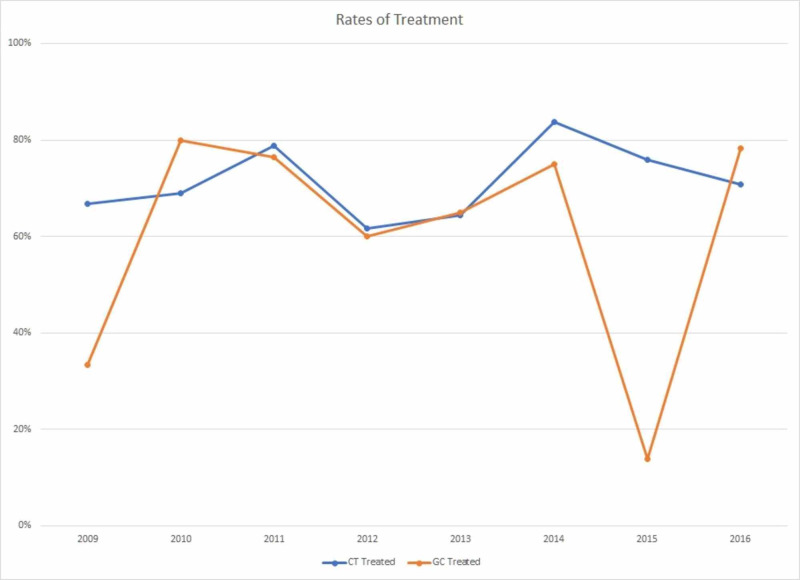
Rate of Chlamydia trachomatis and GC treatment in female adolescent juveniles from 2009 to 2016 CT=Chlamydia; GC=Gonorrhea

Table 1 presents the rates of chlamydia and GC among adolescent females from 2009 to 2016. Among females, screening coverage for CT and GC infections was between 97.9% in 2009 and 99.2% in 2016 (p=0.0016). The rate of CT infections in female adolescents increased from 11.8% in 2009 to 17.0% in 2016 (p=0.0002), with a peak of 19.1% in 2014. An increasing trend of GC infections was also observed, with a rate of 1.3% in 2009 and 6.0% in 2016 (p<0.0001). Treatment for CT was relatively consistent over the study period, at 66.7% in 2009 and 70.8% in 2016 among CT infected patients (p=0.1752). Treatment for GC increased from 33.3% in 2009 to 78.3% in 2016 among GC infected patients, but the overall trends were not statistically significant (p=0.8419). Figure 1 presents the infection rates of CT and GC after screening, indicating a steadily, increasing trend of infection for both diseases. Figure 2 presents the rate of treatment for both CT and GC within the correctional facilities following a positive result. CT treatment remained fairly constant within the time period, whereas the rate of GC treatment dramatically dropped in 2015, most likely due to early release programs.

**Table 1:** Overall rate of Chlamydia trachomatis and GC infections in female adolescent juveniles in San Bernardino County correctional facilities from 2009 to 2016 CT=Chlamydia; GC=Gonorrhea; **p-value was calculated after excluding 2009 and 2015 data.

Intake Year	2009	2010	2011	2012	2013	2014	2015	2016	P-value for trend
Booked	729	593	590	538	537	523	499	385	N/A
Screened	714	582	582	533	532	519	497	382	N/A
CT Infections	84	71	99	81	84	99	87	65	N/A
CT Treated	56	49	78	50	54	83	66	46	N/A
GC Infections	9	15	17	15	20	32	36	23	N/A
GC Treated	3	12	13	9	13	24	5	18	N/A
Rates of disease									
Overall Screening	97.9%	98.2%	98.6%	99.1%	99.1%	99.2%	99.6%	99.2%	0.0016
CT Infections among those who were screened	11.8%	12.2%	17.0%	15.2%	15.8%	19.1%	17.5%	17.0%	0.0002
CT Treated among those who were CT infected	66.7%	69.0%	78.8%	61.7%	64.3%	83.8%	75.9%	70.8%	0.1752
GC Infections among those who were screened	1.3%	2.6%	2.9%	2.8%	3.8%	6.2%	7.2%	6.0%	<0.0001
GC Treated among those who were GC infected *	33.3%	80.0%	76.5%	60.0%	65.0%	75.0%	13.9%	78.3%	0.8419

## Discussion

The current study demonstrates a progressive increasing trend of both CT and GC infections upon booking of female adolescents within the San Bernardino County juvenile detention system. In contrast, a recent study by Burghardt et al. noted a decreasing trend of CT infections among incarcerated female juveniles since the implementation of a CT screening program (CLaSP) in California [9]. Despite the overall statewide success of CLaSP, this study suggests that San Bernardino County infection rates are in contrast to previously reported patterns.

The reason for the increasing prevalence of both CT and GC in adolescent female juveniles in San Bernardino county is likely multifactorial, but may be related to rapid release from the detention center and failure of linkage programs to allow for notification and treatment of infected adolescents. Belenko et al. emphasize the importance of timeliness of screening and also the importance of expanding access to risk reduction and STD prevention programs after release from correctional facilities [7]. We found the number of female adolescent juveniles released prior to notification and treatment of either CT or GC varied from year to year. In the current protocol in San Bernardino County, adolescents released prior to a positive result, which could take seven to twelve days, are notified of their result via a direct call by nursing staff and encouraged to seek treatment. All CT and GC positive cases were reported to the Department of Public Health, but the opportunity for adequate follow-up care may be limited by resource availability. Additionally, most correctional screening programs do not have a linkage pathway to establish care at a community health clinic. As a result, CT and GC positive youth released back into their community are frequently untreated and must independently seek adequate treatment. While county correctional facilities have recognized the importance of timeliness in incorporating screening upon booking, they have yet to fully utilize partnerships to expand access to complimentary programs post release. Another targeted area for collaboration may be community women’s health clinics, which could provide more focused treatment for STDs, education on prevention and potential long-term health risks, and access to a wider range of contraceptive options. Although there are currently some partnerships for re-entry between juvenile correctional facilities and community organizations, there exists an opportunity to expand upon such collaborations and specifically improve access to treatment of STDs within the surrounding communities.

Due to the retrospective design, our study has several limitations. It is possible that some positive screening tests were repeats due to recidivism, both without treatment or with treatment but subsequent reinfection. However, repeat positive results are estimated to make up only 1.6% of positive results and thus are unlikely to skew our findings. Additionally, there was a significant decrease observed in the rate of treatment for adolescents who tested positive for GC in 2015, which may have stemmed from a variety of factors, such as a change in budget allocation, errors in reporting, early release programs, or delayed treatment due to notification following release. Another limitation of the current study is that raw data were collected in an aggregate format and thus we are unable to determine subject level differences such as age, race, or ethnicity disparities.

The disease burden of STDs within the juvenile population is relatively large and costly, with juveniles accounting for nearly 60% of STD cases in San Bernardino County in 2016 [12,13]. While screening programs may seem costly (median cost of $708 per treated individual), the cost-effectiveness over time will likely mitigate initial start-up costs [9,12]. Incorporating screening into the initial medical exam upon booking has been shown to reduce the STD disease burden [14]. Though there are proposed cuts to prevention programs such as CLaSP, such cuts could potentially result in an overall increased cost to government funds, doubling even that of the value of the proposed budget cut [15]. These programs present an opportunity for at-risk youth to receive testing, notification of infection, and medical care which they otherwise would not have access to outside of the correctional system.

Female juveniles account for a significant percentage of the sexually transmitted disease burden due to a number of different factors, including young age which can result in risky behaviors and a disproportionate lack of access to medical resources. Female detainees are also at an increased risk of reinfection due to factors such as infected partners, lack of access to treatment, and proper contraception. Additionally, there is always a risk of treatment failure, particularly if there is a lack of continuity in medical care [15,16]. Sexually transmitted disease may also have significant long-term impacts upon women’s health within the community, including long-term gynecological effects such as pelvic inflammatory disease (PID), infertility, and ectopic pregnancy [17]. These long term impacts can be alleviated with appropriate counseling of STDs and risk reduction programs which have been shown to decrease juvenile STD rates and improve prevention behaviors [12,18]. 

## Conclusions

The persistent high infection rates within the juvenile justice system emphasize the importance of broad screening practices and adequate treatment coverage for chlamydia and GC infections among female adolescents. Additionally, more collaboration between correctional facilities, the public health department, and linkage programs to community medical clinics is needed to improve access to treatment and further ease the burden of STD infection among this vulnerable population.
